# Effects of Oat β-Glucan on the Textural and Sensory Properties of Low-Fat Set Type Pea Protein Yogurt

**DOI:** 10.3390/molecules28073067

**Published:** 2023-03-29

**Authors:** Peiyao Zhao, Nana Li, Lingyun Chen, Yahong Guo, Yatao Huang, Litao Tong, Lili Wang, Bei Fan, Fengzhong Wang, Liya Liu

**Affiliations:** 1 Institute of Food Science and Technology, Chinese Academy of Agricultural Sciences, No. 2 Yuan Ming Yuan West Road, Beijing 100193, China; 2 Agrotechnology & Food Sciences Group, Laboratory of Physics and Physical Chemistry of Foods, Wageningen University, P.O. Box 17, 6700 AA Wageningen, The Netherlands; 3 Department of Agricultural Food & Nutritional Science, University of Alberta, Edmonton, AB T6G 2P5, Canada

**Keywords:** pea protein, low-fat yogurt, oat β-glucan, flavoring substance

## Abstract

This study investigated the effect of oat β-glucan as a fat substitute on the structure formation, texture, and sensory properties of pea protein yogurt. The results showed that the incorporation of 0.5% β-glucan significantly accelerated the lactic acid bacteria-induced fermentation, with the time for reaching the target pH of 4.6 shortened from 3.5 h to 3 h (*p* < 0.05); increased the plastic module (G′) from 693 Pa to 764 Pa when fermenting 3 h (*p* < 0.05); and enhanced the water-holding capacity from 77.29% to 82.15% (*p* < 0.05). The identification of volatile organic compounds (VOCs) in low-fat pea protein yogurt by GC-IMS revealed a significant decrease in aldehydes and a significant increase in alcohols, ketones and acids in the pea yogurt after fermentation (*p* < 0.05). Among them, the levels of acetic acid, acetone, 2,3-butanedione, 3-hydroxy-2-butanone, and ethyl acetate all significantly increased with the addition of oat β-glucan (*p* < 0.05), thereby providing prominent fruity, sweet, and creamy flavors, respectively. Combined with the results of sensory analysis, the quality characteristics of pea protein yogurt with 1% oil by adding 1% oat β-glucan were comparable to the control sample with 3% oil. Therefore, oat β-glucan has a good potential for fat replacement in pea protein yogurt.

## 1. Introduction

With changes in people’s consumption concepts and the increase of vegetarianism, plant-based foods and beverages have become increasingly popular recently, such as plant-based cheese and plant-based milk [1,2]. Among them, fermented plant-based yogurts with lactic acid bacteria have gained high interest because of their beneficial effects on gut health [3]. The rheology and texture of yogurt are critical quality properties that concern consumers. For traditional yogurt, milk fat globules are the primary contributor to hardness and rheology, providing the yogurt with a silky and full-bodied taste through cross-linking with protein [4,5]. During plant protein-based yogurt processing, a high amount of exogenous oil is always added to improve the overall flavor and structure due to the absence of milk fat globules [6,7]. Nevertheless, this is contrary to the consumers’ expectations for consciously reducing fat intake in the daily diet. Therefore, finding suitable fat substitutes to meet consumers’ health needs is very necessary.

β-Glucans are bioactive polysaccharides that are widely distributed in cereals and fungi [8,9]. Cereal is a good source of β-gluten, primally dominant in barley and oats [8]. Oat β-gluten has beneficial physiological activities such as lowering cholesterol, reducing postprandial blood glucose response, preventing diabetes and regulating immunity [10]. Additionally, oat β-glucan is a natural food colloid and thickener with good emulsifying stability and higher viscosity [11]. In the work of Brennan et al. [12], the addition of oat β-glucan to low-fat traditional yogurt significantly improved the viscosity of the product. However, in another work by Qu et al. [13], the addition of oat β-glucan destroyed the three-dimensional network structure of traditional yogurt, and some spherical aggregate particles were observed in the products containing 0.3% β-glucan. Therefore, more research is required to understand the impact of β-glucan on the overall quality of yogurt products as affected by β-glucan levels, as well as the types of yogurts. 

Many kinds of plant material, such as legumes, cereals, pseudocereals, and oil seeds, have been applied to prepare plant-based yogurt [14]. Pea (*Pisum sativum* L.) is one of the most important cultivated legume crops in the world, and its protein is considered to be an emerging alternative protein resource because of its high nutritional value, low cost, low sensitization and non-transgenic status [15]. Pea protein has also demonstrated good functional traits, such as solubility, emulsification, and water-holding capacity [16,17,18]. The use of pea protein in plant yogurt has drawn much attention recently [19,20,21,22]. It has been suggested that the quality of pea protein yogurt is affected by many factors, such as pea protein denaturation, starter cultures, the interactions between pea protein and other components in the systems, and so on [21,22,23]. Nevertheless, the use of oat β-glucan for fat replacement in low-fat, set-style pea protein yogurt has not been reported yet.

The present study aims to evaluate the effects of replacing fat with different amounts of oat β-glucan on the fermentation, gel formation, texture and sensory properties of pea protein yogurt. This study might provide the theoretical basis for the application of oat β-glucan in plant-protein-based yogurt.

## 2. Results and Discussion

### 2.1. The pH of Yogurt

**Figure 1:**
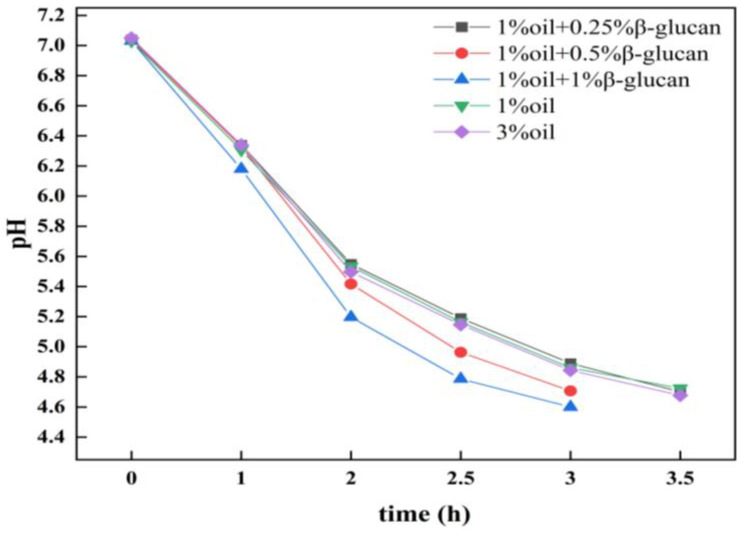
The pH changes of low-fat pea-protein-based yogurt during the fermentation process as affected by oat β-glucan concentration.

Figure 1 shows the effect of oat β-glucan on the fermentation characteristics of low-fat pea protein yogurt. It indicates that, in the absence of oat β-glucan, the fermentation curves of pea protein yogurt prepared with 1% and 3% oil were similar. After 3.5 h of fermentation, their pH values were still higher than the target endpoint (pH 4.6). 

Furthermore, after adding oat β-glucan to pea protein yogurt with 1% fat, it was found that both 0.5% and 1% of oat β-glucan could accelerate the decrease in pH and therefore shorten the fermentation time from 3.5 h to 3 h. The results suggested that 0.5% and 1% oat β-glucan could promote the fermentation performance of lactic acid bacteria in pea protein yogurt, which is consistence with previous studies showing that the addition of oat β-glucan in yogurt could improve the viability and stability of probiotics, and accelerate the production of lactic acid and propionic acid [24].

### 2.2. Rheological Properties

**Figure 2:**
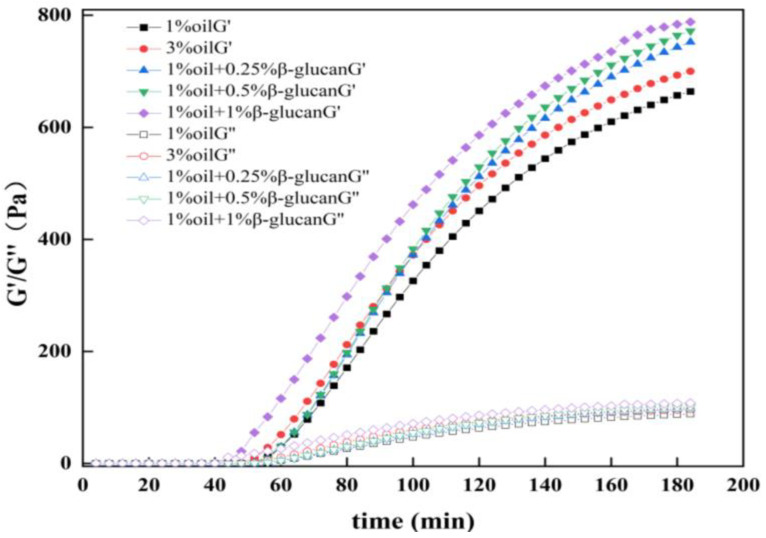
Effects of oat β-glucan concentration on the storage modulus (G′) and loss modulus (G″) of low-fat pea-protein-based yogurt.

Rheology was employed to observe the formation of gel in yogurt. In general, storage modulus (G′) and loss modulus (G″) represented the elastic behavior and viscous behavior of the sample, respectively [21]. As shown in Figure 2, the G′ and G″ values of the yogurt samples increased dramatically after around 40 min of fermentation, and all the G′ values were much higher than the G″ values, exhibiting a typical characteristic of yogurt gel. The fat content was an essential factor in the elasticity of the yogurt, and the reduction of the oil content from 3% to 1% decreased the elasticity of the pea protein yogurt, which was attributed to the weakening of the interactions between oil droplets and protein. The elasticity of yogurt containing oat β-glucan was much higher than that prepared with 3% oil. Additionally, as the concentration of oat β-glucan increased, the elasticity of yogurt was also enhanced. The reason, probably, was that the interaction between pea protein and oat β-glucan was enhanced, which was beneficial to improve the elastic properties of yogurt. The results were consistent with the studies of defatted walnut meal flour/oat β-glucan mixture and silver carp surimi/oat β-glucan mixture [25,26]. 

**Figure 3:**
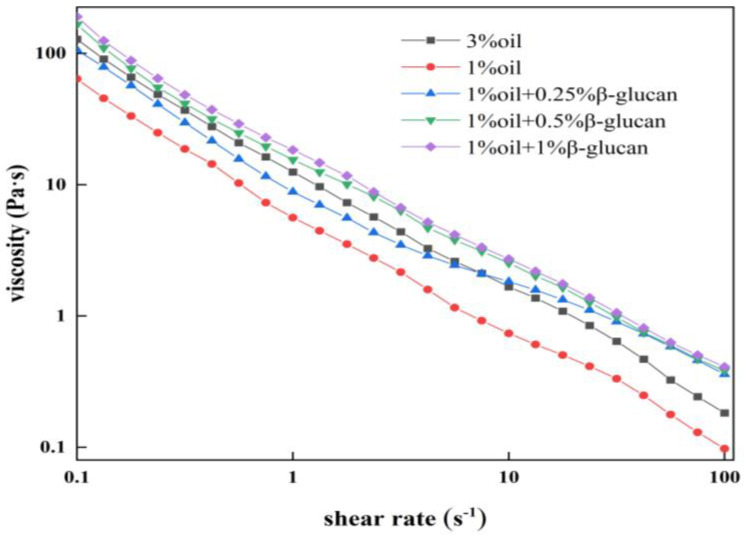
The viscosity of low-fat pea-protein-based yogurt versus shear rate as a function of oat β-glucan concentration.

The apparent viscosity of all yogurt samples decreased with shear rate (Figure 3), indicating a typically shear-thinning fluid behavior [27]. In the absence of oat β-glucan, pea protein yogurt prepared with 1% oil had the lowest apparent viscosity and was positively correlated to the oat β-glucan concentration. According to Figure 2, it was reasonable to assume that the gel structure in the pea protein yogurt could withstand higher shear force, and the three-dimensional network structure became more stable by adding oat β-glucan. Similarly, the addition of inulin to yogurt sharply increased the viscosity of the yogurt [28]. Raikos et al. [29] concluded that hydrophilic polysaccharides had good hydration ability, so water could be locked in the three-dimensional structure, which improved the viscosity of yogurt.

### 2.3. Texture Properties and Water-Holding Capacity

Texture was a desirable indicator for evaluating the quality of yogurt. Table 1 depicts the texture properties of pea protein yogurt as affected by oat β-glucan concentrations. It was clear that the reduction of oil content from 3% to 1% significantly decreased yogurt firmness (*p* < 0.05). When the oat β-glucan concentration increased from 0.25% to 1%, pea protein yogurt firmness improved from 133.58 g to 160.32 g, which was much higher than that of yogurt with 3% oil. A similar trend was observed for chewiness, because it is positively related to firmness. These findings were consistent with the result of rheology properties, which showed the increase in G′ values of the yogurt with the increasing concentration of oat β-glucan. Correspondingly, the water-holding capacity of 1% pea protein yogurt increased significantly with oat β-glucan levels (*p* < 0.05), and the probable reason was that the yogurt added with oat β-glucan formed dense three-dimensional network structures. Wang et al. [30] demonstrated that polysaccharide molecules were a source of hydroxyl groups, which could interact with water molecules through hydrogen bonds, resulting in enhanced water-holding ability. It was also noticed that the springiness and cohesiveness changed little with β-glucan concentration.

### 2.4. SEM Analysis

**Figure 4:**
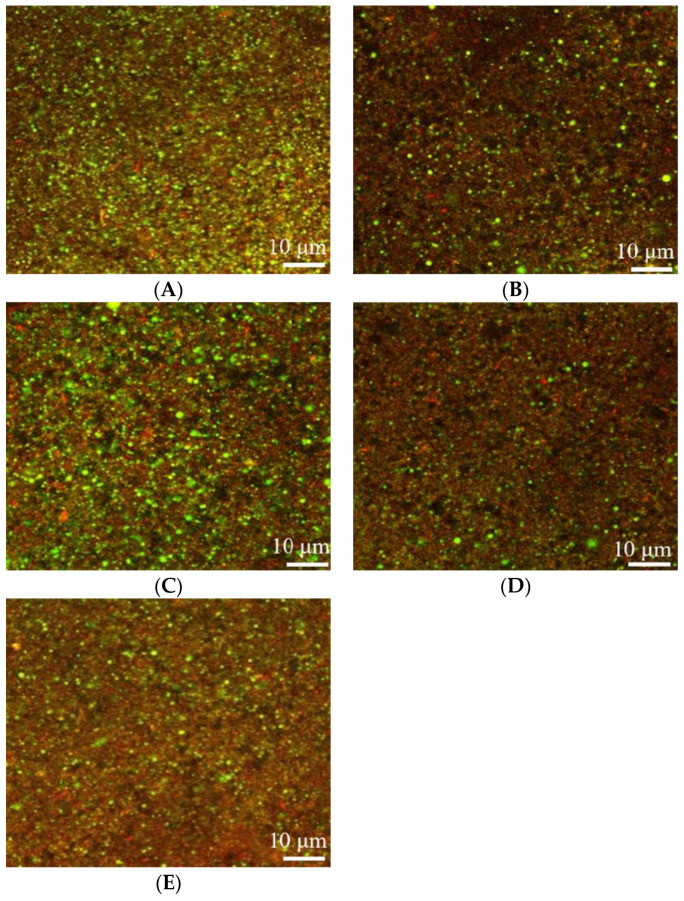
Effects of oat β-glucan concentration on the microstructure of pea-protein-based yogurt: (**A**) yogurt prepared with 3% oil, (**B**) yogurt prepared with 1% oil, (**C**) yogurt prepared with 1% oil and 0.25% oat β-glucan, (**D**) yogurt prepared with 1% oil and 0.5% oat β-glucan, and (**E**) yogurt prepared with 1% oil and 1% oat β-glucan.

The CLSM was used to identify the microstructure of pea protein yogurt (Figure 4). Compared to the yogurt prepared with 3% oil, the pea protein yogurt made with 1% oil exhibited a looser network and had more voids (Figure 4B). As the oat β-glucan concentration increased, the pores in the gel structure became smaller and exhibited a denser gel network structure. This phenomenon might be due to the ability of β-glucan to interact with the protein, thus preventing excessive protein aggregation in a gel, phase separation and the formation of a denser structure. Additionally, β-glucan had high water absorption and thus free water could fill the gel structure, forming a strong three-dimensional structure [31]. Similarly, curdlan gum with a triple helix structure was able to interact with protein molecules, thus strengthening the gel structure [32]. However, Guggisberg et al. [33] found that adding inulin to yogurt did not change the microstructure and morphology of the yogurt gel.

### 2.5. Sensory Evaluation

As shown in Table 2, the reduction of the oil content of pea protein yogurt from 3% to 1% dramatically decreased the sensory properties of the pea protein yogurt (*p* < 0.05). Poor sensory quality was also observed for pea protein yogurt at a low oat β-glucan addition level. However, with the addition of oat β-glucan, the luminosity of yogurt with 1% oil added was enhanced, the sensory properties of it were improved and the firmness of it also increased. The pea protein yogurt with 1% oat β-glucan had the best sensory properties. 

### 2.6. Volatile Flavor Compounds

**Figure 5:**
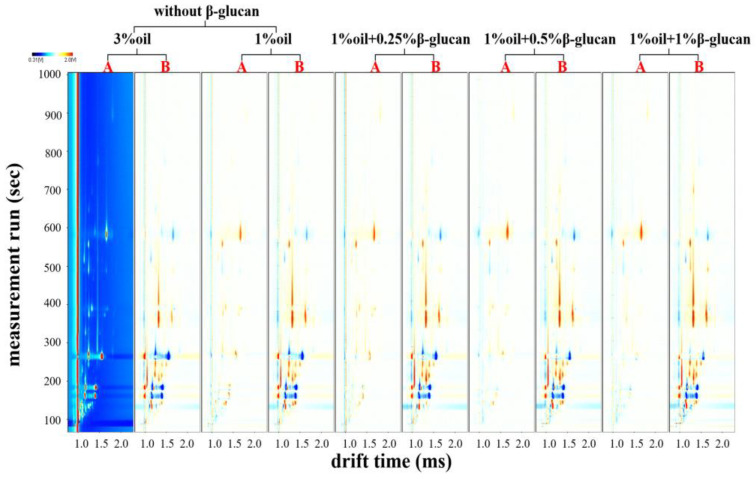
Effect of oat β-glucan concentration on the two-dimensional difference spectra of VOCs before and after fermentation: A and B represent the samples with or without fermentation, respectively.

Gas chromatography–ion mobility spectrometry (GC-IMS) is a novel analytical technique operated at ambient pressure and temperature. It has the advantages of low detection limit, good selectivity and miniaturization [34,35]. In addition, this method can effectively and intuitively display the differences among samples in color-contour images [36]. Figure 5 reflects the effects of oat β-glucan on VOCs in pea protein yogurt before and after fermentation. When 3% oil was used as a background reference against other sample spectra, other samples had more or fewer red and blue spots in their spectra. The red spots showed that the concentration of VOCs was higher than the control, but the blue spots showed the opposite result. From Figure 5, it could be seen that VOCs content in all samples after fermentation was significantly higher than that before fermentation; thus, fermentation facilitated the formation and accumulation of VOCs. Yang et al. [21] also found that VOCs content in pea protein yogurt and mug bean protein yogurt increased after fermentation.

**Figure 6:**
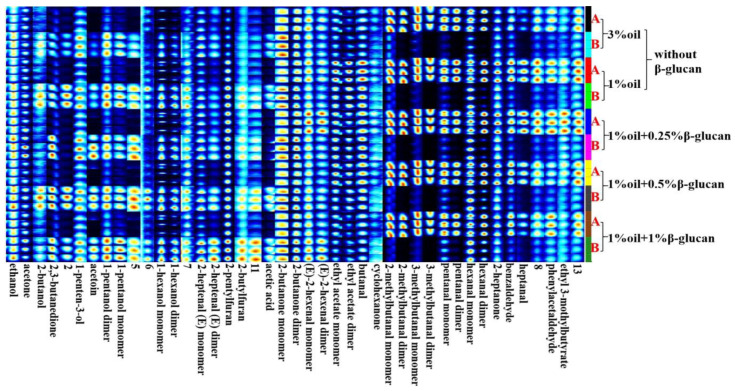
The fingerprints of low-fat pea-protein-based yogurt with different levels of oat β-glucan before and after fermentation: A and B represent the samples with or without fermentation, respectively.

**Figure 7:**
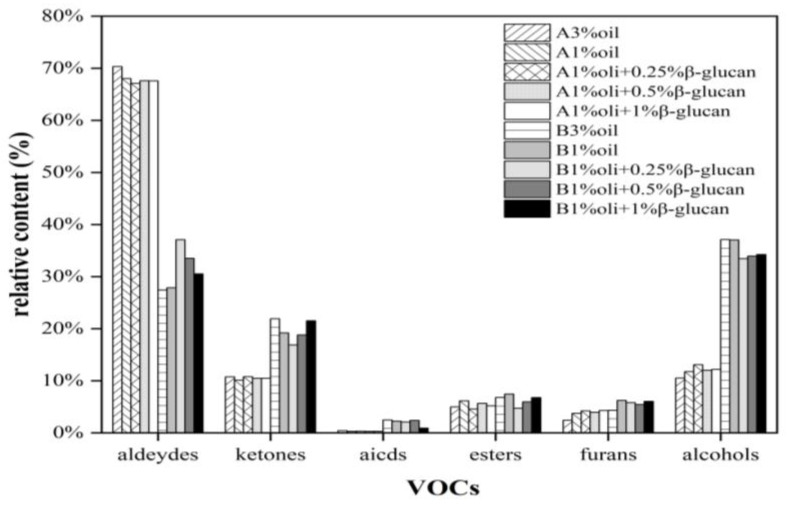
The relative content of pea-protein-based yogurt with different levels of oat β-glucan before and after fermentation: A and B represent the samples with or without fermentation, respectively.

Figure 6 shows the fingerprint profiles of pea protein yogurt samples before and after fermentation using the Gallery Plot plug-in, which exhibited the complete information of VOCs at different oil contents as well as different β-glucan additions and the differences in VOCs. Thirty-eight volatile flavor compounds were detected, including sixteen aldehydes, eleven alcohols, five ketones, three esters, two furans and one acid. These VOCs constituted the characteristic flavor of pea protein yogurt. As can be seen from Figure 3, Figure 4, Figure 5 and Figure 6, region b showed that the peak intensities of (E)-2-hexenal, phenylacetaldehyde, heptanal, benzaldehyde, 2-heptanone, hexanal, pentanal, cyclohexanone and butyraldehyde were significantly stronger before fermentation than after fermentation, and these compounds were the major VOCs in pea milk before fermentation. In contrast, region a showed the opposite peak intensities for acetic acid, 2-n-butylfuran, 2-pentylfuran, 2-heptenal, hexanol, n-pentyl alcohol, 3-hydroxy-2-butanone, 1-penten-3-ol, 2,3-butanedione, acetone and ethanol, which were the main VOCs in pea yogurt after fermentation. To further analyze the effect of fermentation as well as β-glucan addition on VOCs, the relative content of VOCs was calculated using the normalization method, as shown in Figure 7. The results showed that aldehydes content significantly reduced, but the content of alcohols, ketones and acids significantly increased in pea protein yogurt after fermentation compared to VOCs before fermentation (*p* < 0.05).

The effects of oat β-glucan on VOCs in pea protein yogurt are clearly characterized in Table 3, showing the relative content of flavor substances in all samples. 

Hexaldehyde, 1-octen-3-ol and 2-pentylfuran are known to be highly representative flavor substances in soy products. They all had low threshold values, and their contents had a large impact on the overall flavor of the product [37]. Hexanal was closely related to the beany flavor and a grass-like flavor in soymilk [38]. MacLeod et al. [39] found that the threshold value of the sensory detection of hexanal was 19 µg/L in water, so it was important for overall flavor. As shown in Table 2 and Table 3, the hexaldehyde content decreased significantly after fermentation, its dimer decreased from 29.82% to 7.68% in the 3% oil sample, and all other samples also showed a significant decrease. Some studies have shown that the use of Lactobacillus plantarum fermentation of soymilk could improve the flavor of soymilk by significantly reducing the smell of beans and grass. Fatty aldehyde was the main component of soymilk fishy substances, and it could be effectively transformed through the fermentation of Lactobacillus plantarum. This change favored the acceptability of pea yogurt. The 1-octen-3-ol had a mushroom odor [37]. The 1-octen-3-ol content increased after fermentation, and it could be found that 1% oil pea protein yogurt with oat β-glucan added after fermentation possessed higher content than that without the addition of oat β-glucan, thus showing that the addition of oat β-glucan facilitated the accumulation of 1-octen-3-ol. The 2-pentylfuran was produced due to linoleic acid oxidation by the singlet oxygen [40]. The 2-pentylfuran is a product of the oxidation of linoleic acid, and at high concentrations it gives off a bad bean odor and brings a bad flavor to pea protein yogurt. There was a certain increase in its content after fermentation. However, the effect of fermentation on 2-pentylfuran content was much smaller than its effect on hexanal. Overall, fermentation favored the flavor improvement of pea protein emulsions. After fermentation, the content of pentanal dimer decreased from 10.01% to 1.65%, and pentanal had a pungent taste at high concentrations and a nutty taste at low concentrations [41]. In addition, compared to VOCs before fermentation, the content of n-hexanol, 2,3-butanedione, 3-hydroxy-2-butanone, acetone, ethanol and ethyl acetate increased after fermentation. Among them, the content of ethanol, acetone, 2,3-butanedione, 3-hydroxy-2-butanone and ethyl acetate increased significantly with the addition of oat β-glucan. These VOCs provided prominent fruit flavor, a sweet flavor and cream flavor for pea protein yogurt, which was consistent with the sensory evaluation of flavor. When 1% β-glucan was added, the content of 3-hydroxy-2-butanone in pea protein yogurt was the highest (2.34%) and had a strong milk flavor. Therefore, the pea protein yogurt with 1% oat-β-glucan had the best flavor (Table 2).

## 3. Materials and Methods

### 3.1. Materials

Pea protein (protein content 76.91%, N × 6.25) prepared by sour liquid processing was obtained from Shandong Jianyuan Food Co., Ltd. (Yantai, China). Oat β-glucan was kindly provided by Hebei Jinlu Biotechnology Co., Ltd. (Zhangjiakou, China). Sunflower seed oil and sucrose were purchased from Beijing Guangda Hengyi Co., Ltd. (Beijing, China). Commercial yogurt starter VEGE 022 was provided by Danisco Co., Ltd. (Copenhagen, Denmark) and contained *Streptococcus thermophilus*, *Lactobacillus delbrueckii subsp. bulgaricus*, *Lactobacillus plantarum*, *Lactobacillus acidophilus* (NCFM^®^) and *Bifidobacterium lactis* (HN019™). Nile Red was purchased from Beijing Solebo Biotechnology Co., Ltd. (Beijing, China). Nile blue was purchased from Sigma-Aldrich (St. Louis, MO, USA). 

### 3.2. Yogurt Preparation

Stock solutions of oat β-glucan (3.0%/*w*/*v*) and pea protein (5.0%/*w*/*v*) were prepared in distilled water by stirring at room temperature until the samples were fully hydrated. The mixtures containing 3% pea protein and different amounts of β-glucan (0–1%) were prepared by mixing the appropriate volume of each concentrated solution up to the required concentration After that, the sucrose (5%/*w*/*v*) and sunflower oil (1%/*w*/*v*) were added to the mixtures and sheared at 10,000 r/min using a high-speed disperser for 2 min. The dispersions were then homogenized at 40 MPa, and homogenized pea protein milk was heated at 85 °C for 20 min and cooled to 40 °C. The pea protein milk samples were inoculated with starters (1 g/L VEGE 022) and fermented at 37 °C until the pH reached 4.6. The samples were stored at 4 °C in a refrigerator for 24 h for further analysis. Yogurt containing 3% oil in the absence of β-glucan was also prepared as a control. 

### 3.3. Measurement of pH

The pH of the yogurt samples was determined every 30 min using a pH meter (FE28, Mettler-Toledo, Zurich, Switzerland).

### 3.4. Rheological Properties

The rheological measurement was taken by a rheometer (MCR 301, Anton Paar, Graz, Austria).

Time sweep: inoculated pea protein milk samples were put into a cylinder at 37 °C for fermentation, and the pH was monitored using a pH meter. When the pH values of the yogurt reached the endpoint (4.6), the time sweep stopped. The time sweep test was performed at a fixed frequency of 1 Hz. The storage modulus (G′) and the loss modulus (G”) of the yogurt were determined during the fermentation. 

Frequency sweep: after the time sweep, the frequency sweep was performed. The temperature was cooled down to 4 °C at a cooling rate of 1 °C/min, and the yogurt was stored for 30 min. The frequency sweep was carried out within the range of 0.01 to 10 Hz at a fixed strain of 0.1%, and the storage modulus (G′) and loss modulus (G”) of the yogurt were determined. 

### 3.5. Texture Characteristics

The textural characteristics of yogurt were measured using a texture analyzer (TA, TAXT2i, Stable Micro System, Godalming, UK) equipped with a P/36R probe (36 mm). The speeds of the pre-test, test, and post-test were set at 1.0 mm/s, and the strain was 30%. All yogurt samples were stored at 4 °C and allowed to stand for 24 h before texture tests. 

### 3.6. Water-Holding Capacity

The water-holding capacity (WHC) was determined. Inoculated pea protein milk (20 g) was fermented in a 50 mL centrifugal tube. After fermentation, the pea protein yogurt was stored at 4 °C for 24 h. Then, the yogurt sample was centrifuged for 20 min, the supernatant was removed and the remaining pea protein yogurt was weighed. The WHC of yogurt was calculated using the following formula:

(1) $$WHC=\frac{m_{1}}{m_{2}}\times 100$$

where m_1_ is the weight of the remaining yogurt, and m_2_ is the total weight of the sample.

### 3.7. Microstructure 

Confocal laser scanning microscopy (CLSM) was performed to obverse the microstructure of the yogurt. Before observation, 1 mL inoculated pea protein milk was stained with 20 uL Nile Blue (0.1%/*w*/*v*) for 5 min. The stained samples were transferred to a glass slide, covered with a coverslip and fermented at 37 °C until pH reached 4.6. Then, they were stored at 4 °C and allowed to stand for 24 h. The yogurt samples were observed with a CLSM (880LSM, Carl Zeiss AG, Oberkochen, Germany) with a 40× objective lens. The excitation wavelength was set at 638–759 nm and all images were captured in 1024 × 1024 pixels.

### 3.8. Sensory Evaluation 

According to GB/T30885-2014, sensory evaluation Table 4 was made. Twelve students (six men and six women, aged 20–30) majoring in food and having some experience in sensory evaluation were invited to form a sensory evaluation team. Pea protein yogurt after 24 h post-ripening was placed in coded yogurt cups for the sensory team to taste.

### 3.9. Determination of Volatile Flavor Compounds 

Volatile flavor compounds (VOCs) of pea protein yogurt were analyzed by gas-phase ion mobility spectrum FlavourSpec^®^ (the G.A.S. Department of Shandong Hai Neng Science Instrument Co., Ltd., Dezhou, China). The testing conditions were as follows.

The yogurt samples (5 mL) were added to the 20 mL headspace glass and sealed. Then, the sample was inserted into the sampling tank with an automatic sampler and incubated at 37 °C for 20 min. Additionally, the injection volume was 500 μL, and the injection needle temperature was 85 °C.

The samples flowed through the MXT-5 capillary column (15 m × 0.53 mm), and the capillary column temperature was 60 °C.

The initial rate of carrier gas was 2 mL/min in 2 min, the rate linearly increased to 10 mL/min in 8 min, and finally the rate increased to 100 mL/min in 10 min.

The temperature of the IMS was 45 °C, the drift gas (E1) and the carrier gas (E2) were nitrogen (purity ≥ 99.999%), and the drift gas flow rate was 150 mL/min.

### 3.10. Statistical Analysis

All experiments were performed in triplicate in all samples. The results were expressed as mean ± standard deviation. The data were analyzed by one-way analysis of variance (ANOVA) followed by Duncan’s multiple range test for post hoc analysis. (*p* < 0.05). Origin 8.0 (Origin Lab, Northampton, MA, USA) was used to draw and analyze. Reporter and Gallery plot plugins in Laboratory Analytical Viewer (LAV) were used to compare volatile flavor compounds.

## 4. Conclusions

An appropriate amount of oat β-glucan (1%) in yogurt could shorten the fermentation time and improve the water-holding capacity, texture and flavor of low-fat pea protein yogurt. Obvious differences in the types and concentrations of VOCs before and after fermentation were observed. After fermentation, aldehydes in pea protein yogurt decreased significantly, while alcohols, ketones and acids increased dramatically (*p* < 0.05). Due to the reduced production of beany flavor compounds, the flavor of yogurt was improved significantly after fermentation. In addition, the content of ethanol, acetone, 2,3-butanedione and 3-hydroxy-2-butanone increased significantly with the addition of β-glucan, which provided a prominent fruit flavor, sweet flavor and milk flavor for pea protein yogurt, respectively. In conclusion, oat β-glucan can be used as a suitable fat replacer in low-fat pea protein yogurt. It would be worthwhile to further study the functional properties of low-fat pea yogurt with oat β-glucan, such as intestinal probiotic properties and auxiliary hypoglycemic activity.

## Figures and Tables

**Table 1:** Effects of β-glucan concentration on texture properties and water holding capacity of low-fat pea-protein-based yogurt.

Pea Protein Yogurt	Firmness/g	Springiness/mm	Cohesiveness	Chewability/g	Water-Holding Capacity/%
3% oil	147.06 ± 4.94 ^b^	98.44 ± 0.18 ^ab^	0.45 ± 0.10 ^d^	64.52 ± 1.79 ^b^	77.29 ± 1.61 ^bc^
1% oil	121.80 ± 1.80 ^d^	98.78 ± 0.55 ^ab^	0.48 ± 0.10 ^a^	58.57 ± 1.49 ^c^	62.60 ± 5.83 ^d^
1% oil + 0.25% β-glucan	133.58 ± 1.76 ^c^	99.09 ± 0.26 ^a^	0.47 ± 0.10 ^ab^	61.90 ± 0.68 ^b^	68.28 ± 7.45 ^c^
1% oil + 0.5% β-glucan	138.28 ± 3.41 ^c^	99.10 ± 0.12 ^a^	0.46 ± 0.10 ^bc^	65.11 ± 2.30 ^b^	82.15 ± 1.01 ^ab^
1% oil + 1% β-glucan	160.32 ± 4.64 ^a^	99.10 ± 0.14 ^a^	0.46 ± 0.10 ^bc^	70.78 ± 1.69 ^a^	87.11 ± 0.41 ^a^

Data are mean ± standard deviation (*n* = 3). Different letters represent significant difference (*p* < 0.05) as determined by one way ANOVA, followed by Duncan’s Test.

**Table 2:** Effects of oat β-glucan concentration on the sensory properties of pea-protein-based yogurt.

Pea Protein Yogurt	Color	Flavor	Taste	Texture	Total Points
3% oil	16.82 ± 0.12 ^a^	9.58 ± 0.22 ^c^	11.21 ± 0.82 ^c^	30.58 ± 0.24 ^c^	70.73 ± 2.97 ^b^
1% oil	6.22 ± 0.24 ^e^	8.56 ± 0.15 ^d^	10.18 ± 0.37 ^d^	15.32 ± 0.11 ^e^	41.42 ± 1.41 ^d^
1% oil + 0.25% β-glucan	10.46 ± 0.85 ^d^	10.70 ± 0.35 ^b^	11.59 ± 0.27 ^bc^	25.16 ± 0.31 ^d^	55.31 ± 4.60 ^c^
1% oil + 0.5% β-glucan	12.80 ± 0.13 ^c^	11.29 ± 0.46 ^b^	12.32 ± 0.56 ^b^	33.21 ± 0.23 ^b^	68.54 ± 2.21 ^b^
1% oil + 1% β-glucan	14.46 ± 0.17 ^b^	15.19 ± 0.51 ^a^	15.89 ± 0.43 ^a^	38.77 ± 0.36 ^a^	82.77 ± 1.67 ^a^

Data are mean ± standard deviation (*n* = 3). Different letters represent significant difference (*p* < 0.05) as determined by one way ANOVA, followed by Duncan’s Test.

**Table 3:** The relative content of volatile flavor compounds before and after fermentation.

Volatile Flavor Compounds	Before Fermentation					After Fermentation				
	3% Oil	1% Oil	1% Oil + 0.25% β-Glucan	1% Oil + 0.5% β-Glucan	1% Oil + 1% β-Glucan	3% Oil	1% Oil	1% Oil + 0.25% β-Glucan	1% Oil + 0.5% β-Glucan	1% Oil + 1% β-Glucan
(E)-2-Hexenal	1.23	1.09	1.06	1.01	1.24	1.75	1.33	1.36	1.3	1.4
2-Heptenal	3.58	2.52	2.49	2.24	3.39	7.64	5.45	6.52	6.06	6.26
2-Methyl-Butanal	8.42	8.57	8.99	9.14	8.77	3.05	2.08	1.37	1.73	1.96
Isovaleraldehyde	7.04	6.72	6.6	6.82	6.63	1.08	0.82	0.75	0.77	0.89
Benzaldehyde	1.91	2.24	2.28	2.24	2.07	1.77	1.43	1.02	1.19	1.23
Butyraldehyde	1.21	1.54	1.09	1.40	1.33	1.51	1.68	0.96	1.26	1.74
Heptaldehyde	0.33	0.58	0.45	0.53	0.63	0.17	0.15	0.16	0.17	0.15
Hexaldehyde	29.82	29.22	29.17	29.7	29.17	7.68	12	20.65	17.36	13.49
Pentanal	16.44	15.25	14.58	14.25	14.01	2.51	2.74	4.09	3.47	3.23
Phenylacetaldehyde	0.34	0.30	0.38	0.27	0.33	0.27	0.17	0.22	0.20	0.19
n-Hexanol	4.66	6.76	7.44	7.01	7.2	24.09	26.22	24.67	24.57	24.48
n-Pentanol	1.27	1.3	1.52	1.14	1.16	5.28	4.71	4.09	3.95	4.08
1-Pentene-3-Ol	0.66	0.55	0.64	0.59	0.61	1.28	0.98	0.82	0.93	1.01
2-Methylpropanol	0.07	0.07	0.07	0.07	0.06	0.17	0.16	0.10	0.14	0.09
2-Methylpropanol	3.84	3.02	3.48	3.15	3.19	5.85	4.16	3.81	3.9	4.63
Ethanol	2.07	1.79	2.09	1.97	2.00	3.64	2.95	2.36	2.65	2.98
Isopropanol	1.77	1.28	1.31	1.22	1.14	2.65	2.02	1.38	1.69	1.58
2,3-Butanedione	1.42	1.26	1.47	1.41	1.33	5.62	5.29	5.36	6.00	6.45
2-Heptanone	2.66	3.06	2.77	3.14	3.07	3.65	3.54	2.57	3.04	3.53
3-Hydroxy-2-Butanone	0.09	0.10	0.17	0.17	0.14	1.22	1.62	1.76	2.00	2.34
Acetone	3.11	2.42	2.65	2.36	2.50	5.24	4.28	3.12	3.59	4.25
Cyclohexanone	0.25	0.25	0.23	0.25	0.26	0.33	0.30	0.25	0.27	0.33
2-n-Butylfuran	0.16	0.14	0.16	0.15	0.16	0.21	0.20	0.21	0.17	0.19
2-Pentylfuran	2.27	3.59	4.05	3.78	4.13	4.10	6.02	5.58	5.25	5.86
Ethanol	0.40	0.30	0.32	0.30	0.30	2.47	2.25	2.09	2.38	2.91
Ethyl Isovalerate	0.29	0.31	0.28	0.33	0.38	0.35	0.28	0.27	0.27	0.39
Ethyl Acetate	4.68	5.8	4.27	5.34	4.8	6.43	5.55	6.09	5.69	6.38

**Table 4:** Sensory evaluation scoring index and criteria of yogurt samples.

Indicator	Total Points	Marking Criteria
Color	20	Uniform color, overall creamy white: 12–20
		Uniform color, creamy yellow color: 4–11
		Uneven color, colored spots, abnormal colors: 0–3
Flavor	20	Natural fermented flavor, no beany flavor, and grass-like flavor: 12–20
		Mild or intense fermented flavor, slight beany flavor and grass-like flavor: 4–11
		Strong beany flavor and grass-like flavor: 0–3
Taste	20	Suitable sour and sweet taste lubricated: 12–20
		Moderately sweet and sour, slightly astringent: 4–1
		Too sour or too sweet, strongly astringent: 0–3
Texture	40	Smooth surface, no whey separation: 31–40
		Smooth surface, small amount of whey separation: 21–30
		The surface is not smooth, a small amount of whey separation: 5–20

## Data Availability

Data are contained within the article.

## References

[1] Mattice K.D., Marangoni A.G. (2020). Physical properties of plant-based cheese products produced with zein. Food Hydrocoll..

[2] Vogelsang-O’Dwyer M., Zannini E., Arendt E.K. (2021). Production of pulse protein ingredients and their application in plant-based milk alternatives. Trends Food Sci. Technol..

[3] Mazahreh A.S., Ershidat O.T.M. (2009). The benefits of lactic acid bacteria in yogurt on the gastrointestinal function and health. Pak. J. Nutr..

[4] Santiago-García P.A., Mellado-Mojica E., León-Martínez F.M., Dzul-Cauich J.G., López M.G., García-Vieyra M.I. (2021). Fructans (agavins) from Agave angustifolia and Agave potatorum as fat replacement in yogurt: Effects on physicochemical, rheological, and sensory properties. LWT Food Sci. Technol..

[5] Mary P.R., Mutturi S., Kapoor M. (2022). Non-enzymatically hydrolyzed guar gum and orange peel fibre together stabilize the low-fat, set-type yogurt: A techno-functional study. Food Hydrocoll..

[6] Levy R., Okun Z., Shpigelman A. (2022). Utilizing high-pressure homogenization for the production of fermented plant-protein yogurt alternatives with low and high oil content using potato protein isolate as a model. Innov. Food Sci. Emerg. Technol..

[7] Chen X., He Z., He L., Li C., Tao H., Wang X., Liu L., Zeng X., Ran G. (2023). Effects of perilla seed oil addition on the physicochemical properties, sensory, and volatile compounds of potato blueberry flavored yogurt and its shelf-life prediction. LWT Food Sci. Technol..

[8] Han X., Luo R., Ye N., Hu Y., Fu C., Gao R., Fu S., Gao F. (2022). Research progress on natural beta-glucan in intestinal diseases. Int. J. Biol. Macromol..

[9] Cui Y., Han X., Huang X., Xie W., Zhang X., Zhang Z., Yu Q., Tao L., Li T., Li S. (2023). Effects of different sources of β-glucan on pasting, gelation, and digestive properties of pea starch. Food Hydrocoll..

[10] Liu B., Lin Q., Yang T., Zeng L., Shi L., Chen Y., Luo F. (2015). Oat beta-glucan ameliorates dextran sulfate sodium (DSS)-induced ulcerative colitis in mice. Food Funct..

[11] Shah A., Gani A., Masoodi F.A., Wani S.M., Ashwar B.A. (2017). Structural, rheological and nutraceutical potential of β-glucan from barley and oat. Bioact. Carbohydr. Diet. Fibre.

[12] Brennan C.S., Tudorica C.M. (2008). Carbohydrate-based fat replacers in the modification of the rheological, textural and sensory quality of yoghurt: Comparative study of the utilisation of barley beta-glucan, guar gum and inulin. Int. J. Food Sci. Technol..

[13] Qu X., Nazarenko Y., Yang W., Nie Y., Zhang Y., Li B. (2021). Effect of Oat beta-Glucan on the Rheological Characteristics and Microstructure of Set-Type Yogurt. Molecules.

[14] Kong X., Xiao Z., Du M., Wang K., Yu W., Chen Y., Liu Z., Cheng Y., Gan J. (2022). Physicochemical, Textural, and Sensorial Properties of Soy Yogurt as Affected by Addition of Low Acyl Gellan Gum. Gels.

[15] Pillai P.K.S., Morales-Contreras B.E., Wicker L., Nickerson M.T. (2020). Effect of enzyme de-esterified pectin on the electrostatic complexation with pea protein isolate under different mixing conditions. Food Chem..

[16] Ge J., Sun C.X., Corke H., Gul K., Gan R.Y., Fang Y. (2020). The health benefits, functional properties, modifications, and applications of pea (*Pisum sativum* L.) protein: Current status, challenges, and perspectives. Compr. Rev. Food Sci. Food Saf..

[17] Guler-Akin M.B., Avkan F., Akin M.S. (2021). A novel functional reduced fat ice cream produced with pea protein isolate instead of milk powder. J. Food Process. Preserv..

[18] Liao W., Fan H., Liu P., Wu J. (2019). Identification of angiotensin converting enzyme 2 (ACE2) up-regulating peptides from pea protein hydrolysate. J. Funct. Foods.

[19] Klost M., Drusch S. (2019). Structure formation and rheological properties of pea protein-based gels. Food Hydrocoll..

[20] Klost M., Giménez-Ribes G., Drusch S. (2020). Enzymatic hydrolysis of pea protein: Interactions and protein fractions involved in fermentation induced gels and their influence on rheological properties. Food Hydrocoll..

[21] Yang M., Li N., Tong L., Fan B., Wang L., Wang F., Liu L. (2021). Comparison of physicochemical properties and volatile flavor compounds of pea protein and mung bean protein-based yogurt. LWT Food Sci. Technol..

[22] Li N., Yang M., Guo Y., Tong L.-T., Wang Y., Zhang S., Wang L., Fan B., Wang F., Liu L. (2022). Physicochemical properties of different pea proteins in relation to their gelation ability to form lactic acid bacteria induced yogurt gel. LWT Food Sci. Technol..

[23] Ma W., Zhang C., Kong X., Li X., Chen Y., Hua Y. (2021). Effect of pea milk preparation on the quality of non-dairy yoghurts. Food Biosci..

[24] Vasiljevic T., Kealy T., Mishra V.K. (2007). Effects of beta-glucan addition to a probiotic containing yogurt. J. Food Sci..

[25] Zhang X., Cheng Z., Zhao X., Liu H., Hu H., Wang M., Guo J. (2022). Effects of the oat β-glucan on the functional and structural properties of defatted walnut meal flour. Food Chem. Adv..

[26] He X., Lv Y., Li X., Yi S., Zhao H., Li J., Xu Y. (2023). Effect of oat β-glucan on gel properties and protein conformation of silver carp surimi. J. Sci. Food Agric..

[27] Wang X., Kristo E., LaPointe G. (2020). Adding apple pomace as a functional ingredient in stirred-type yogurt and yogurt drinks. Food Hydrocoll..

[28] Crispín-Isidro G., Lobato-Calleros C., Espinosa-Andrews H., Alvarez-Ramirez J., Vernon-Carter E.J. (2015). Effect of inulin and agave fructans addition on the rheological, microstructural and sensory properties of reduced-fat stirred yogurt. LWT Food Sci. Technol..

[29] Raikos V., Grant S.B., Hayes H., Ranawana V. (2018). Use of beta-glucan from spent brewer’s yeast as a thickener in skimmed yogurt: Physicochemical, textural, and structural properties related to sensory perception. J. Dairy Sci..

[30] Wang J., Liu B., Qi Y., Wu D., Liu X., Liu C., Gao Y., Shi J., Fang L., Min W. (2022). Impact of Auricularia cornea var. Li polysaccharides on the physicochemical, textual, flavor, and antioxidant properties of set yogurt. Int. J. Biol. Macromol..

[31] Aljewicz M., Majcher M., Nalepa B. (2020). A Comprehensive Study of the Impacts of Oat beta-Glucan and Bacterial Curdlan on the Activity of Commercial Starter Culture in Yogurt. Molecules.

[32] Wu C., Yuan C., Chen S., Liu D., Ye X., Hu Y. (2015). The effect of curdlan on the rheological properties of restructured ribbonfish (Trichiurus spp.) meat gel. Food Chem..

[33] Guggisberg D., Cuthbert-Steven J., Piccinali P., Bütikofer U., Eberhard P. (2009). Rheological, microstructural and sensory characterization of low-fat and whole milk set yoghurt as influenced by inulin addition. Int. Dairy J..

[34] Yang Y., Chen J., Jiang Y., Qian M.C., Deng Y., Xie J., Li J., Wang J., Dong C., Yuan H. (2022). Aroma dynamic characteristics during the drying process of green tea by gas phase electronic nose and gas chromatography-ion mobility spectrometry. LWT Food Sci. Technol..

[35] Yao W., Cai Y., Liu D., Chen Y., Li J., Zhang M., Chen N., Zhang H. (2022). Analysis of flavor formation during production of Dezhou braised chicken using headspace-gas chromatography-ion mobility spec-trometry (HS-GC-IMS). Food Chem..

[36] Wen R., Kong B., Yin X., Zhang H., Chen Q. (2022). Characterisation of flavour profile of beef jerky inoculated with different autochthonous lactic acid bacteria using electronic nose and gas chromatography-ion mobility spectrometry. Meat Sci..

[37] Yuan S.H., Chang S.K. (2007). Selected odor compounds in cooked soymilk as affected by soybean materials and direct steam injection. J. Food Sci..

[38] Ma H., Guan C.-Y., He X.-L., Zhang G.-Z., Din A.L. (2002). Effects of lipoxygenase null genes of soybean in controlling beany-flavor of soymilk and soyflour. Agric. Sci. China.

[39] MacLeod G., Ames J. (1988). Soy flavor and its improvement. Crit. Rev. Food Sci. Nutr..

[40] Min S., Yu Y., Yoo S., Martin S.S. (2005). Effect of Soybean Varieties and Growing Locations on the Flavor of Soymilk. J. Food Sci..

[41] Öztürk H.İ., Aydın S., Sözeri D., Demirci T., Sert D., Akın N. (2018). Fortification of set-type yoghurts with Elaeagnus angustifolia L. flours: Effects on physicochemical, textural, and microstructural characteristics. LWT Food Sci. Technol..

